# Picture Perfect Precision: Biorthogonal Photoactivatable Tools Achieve Imaging with Molecular-Scale Precision

**DOI:** 10.1021/acscentsci.3c00945

**Published:** 2023-08-15

**Authors:** Kai Kikuchi, Amandeep Kaur

**Affiliations:** † Medicinal Chemistry, Monash Institute of Pharmaceutical Sciences, Monash University, Parkville, VIC 3052, Australia; ‡ Australian Research Council Centre of Excellence for Innovations in Peptide and Protein Science, Monash University, Melbourne 3800, Australia

The Nobel Prize is the most prestigious honor for exceptional achievements in a given field. Imagine the possibilities when two Nobel Prize-winning technologies join forces! In this issue of *ACS Central Science*, Hell and colleagues harness the synergistic power of two techniques awarded the Nobel Prize in chemistry–super-resolution microscopy (2014) and biorthogonal labeling (2022)—to achieve optical imaging with molecular-scale precision. Their work describes the development of a family of probes containing both a fluorescence quenching tetrazine for bioorthogonal labeling and a photoactivatable xanthone, allowing for minimal-linkage-error, near-background-free imaging.^1^

The development of fluorescence nanoscopy (otherwise known as super-resolution microscopy, or SRM) has enabled the imaging of biological processes and structures whose details were previously obscured by the diffraction limit of light. A great many techniques have been developed over the years for nanoscale imaging, each with their own pros and cons.^2^ MINimal photon FLUXes (MINFLUX) is one of the few techniques that has enabled imaging at resolutions down to 1 nm.^3^ At such small scales, the choice of fluorophore can significantly hinder the obtainable resolution. Two such fluorophore-associated factors are background fluorescence and linkage error.^4^ Linkage error causes a reduction in resolution due to the length of a linker giving significant spatial separation between target and fluorophore. It is therefore prudent to reduce the linker length where possible.

The use of bioorthogonal labeling strategies such as strain-promoted inverse electron demand Diels–Alder cycloadditions (SPIEDAC) with tetrazines allows for minimal linkage error labeling of biological targets. Tetrazines are also able to effectively quench fluorescence via through-bond energy transfer (TBET) and Förster resonance energy transfer (FRET), resulting in a fluorescence turn on upon reaction.^5^ This property makes them ideal for reducing background fluorescence as only fluorophores attached to the target will be emissive, improving the signal-to-noise ratio. When used in this manner, tetrazines (and other fluorescence quenchers such as azides and *N*-nitrosyl groups) are known as caging groups.

Recently, the **p**hoto**a**ctivatable **x**anthone (**PaX**) fluorophores were developed by the Hell group.^6^ These are notable among fluorogenic fluorophores as they are caging-group-free and do not release any byproducts upon photouncaging. This is because they feature a xanthone core which is nonemissive at the wavelengths imaged, which upon irradiation can undergo radical cyclization with a nearby alkene, forming the fluorescent pyronine.

**Figure 1:**
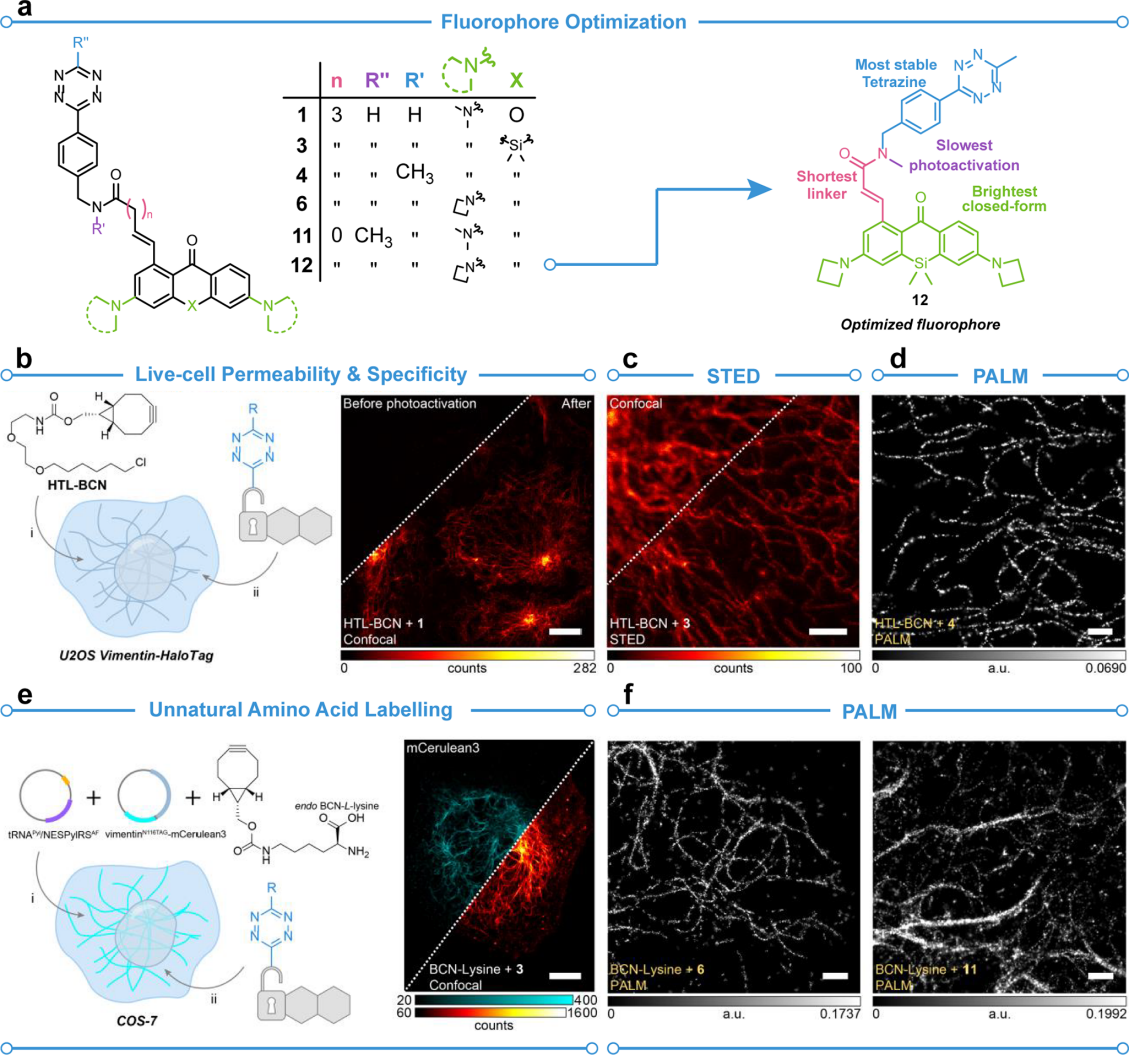
Confirmation of cell-permeability, and direct labeling with **PaX-Tz** fluorophores. (a, left) General structures of **PaX-Tz** fluorophores used in panels b–f. (a, right) **12** was determined to have optimal properties for imaging. (b, left) Live-cell permeability and specificity of BCN labeling were investigated in vimentin-HaloTag constructs expressed by U2OS cells. (b, right) Confocal image taken with compound **1** before and after photoactivation. (c) Comparison of confocal and STED images taken with compound **3**. (d) PALM image taken with compound **4**. (e, left) COS-7 cell expressing a vimentin-mCerulean3 construct incorporating N116TAG mutation encoding for *endo*-BCN-l-lysine was labeled with **PaX-Tz** probes and imaged. (E, right) Confocal image showing an mCerulean signal (cyan) and a compound **3** signal (red). (f) PALM image taken with compounds **6** (left) and **11** (right). Reproduced with permission from ref (1). Copyright 2023 American Chemical Society.

In new work published in *ACS Central Science*, Hell and co-workers combine both **PaX** and tetrazine strategies to form new **PaX** tetrazine (**PaX-Tz**) dyes used in MINFLUX nanoscopy with both minimal background and linkage error. A range of both previously reported and novel **PaX** fluorophores with tetrazines were screened for four properties: most photostable tetrazine, slowest photoactivation, shortest linker, and brightest closed form (**PaX**_**CF**_**-Pz**) (Figure 1a,b). It was found that compound **12** featured the optimal properties. **PaX 12** contained a dimethylsilicon bridge (greater quenching of photoactivation than an O-bridge), an azetidine auxochrome (higher closed-form fluorescence quantum yield [φ_f_] than for dimethylamine), a tertiary acrylamide linker (shortest linker, allowing for reduced linkage error, and slowest photoactivation, allowing greater control during imaging), and a methyl tetrazine (highest bench stability). Notably, the activated silicon **PaX**_**CF**_**-Pz** fluorophores have significantly blue-shifted absorption maxima (λ_abs_ ≈ 570–580 nm) compared to their silicon-rhodamine counterparts (λ_abs_ ≈ 640–650 nm).^7^

**Figure 2:**
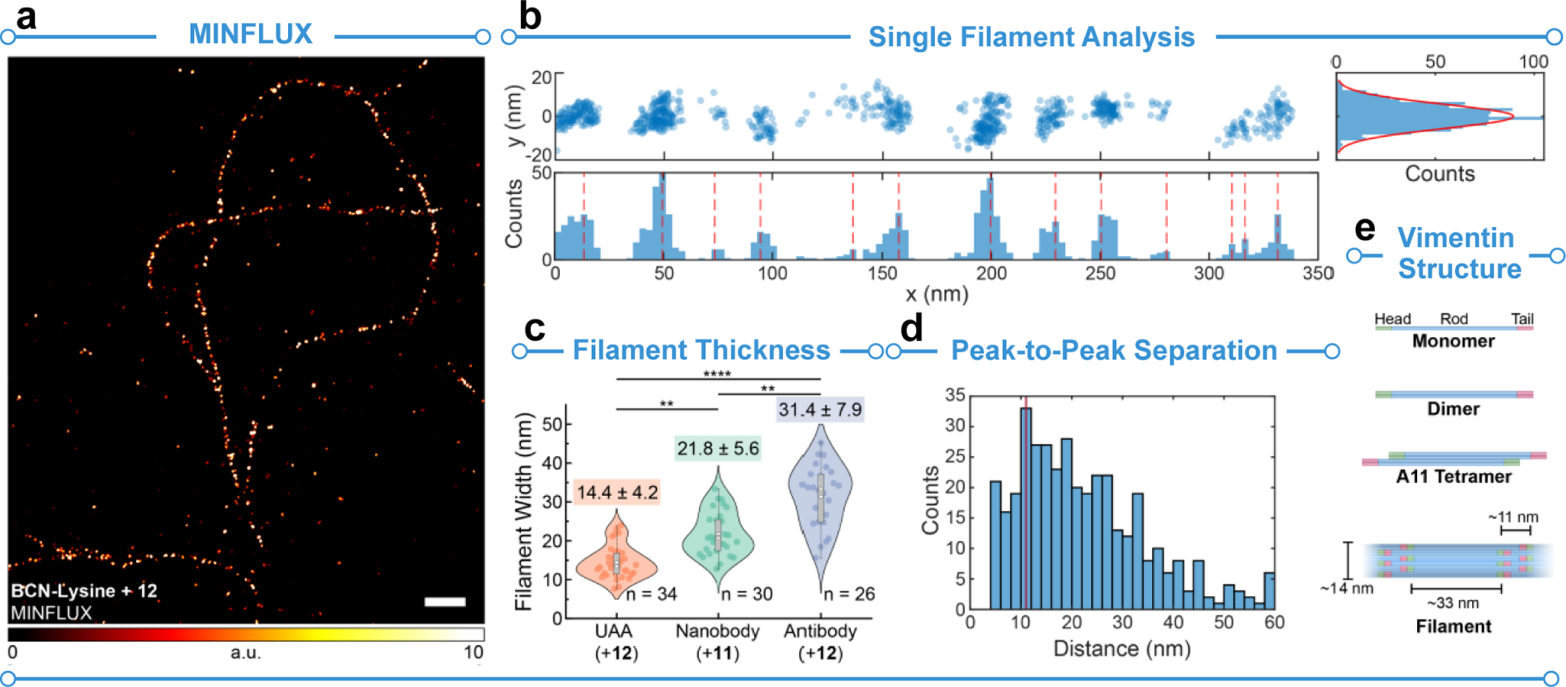
MINFLUX imaging and characterization of vimentin labeled with compound **12**. (a) MINFLUX image of vimentin N116TAG encoding for *endo*-BCN-*L*-lysine labeled with compound **12**. (b) Single filament analysis of the filament shown in a. Shown is the distribution of localizations along the length (*x*) and width (*y*). These data were used to determine filament thickness (c) and peak-to-peak separation (d). (c) Violin plot showings filament thickness distributions of vimentin labeled with UAA incorporation, nanobody, and antibody strategies. (d) Peak-to-peak separations as calculated from b. (e) Structure of vimentin. Reproduced with permission from ref (1). Copyright 2023 American Chemical Society.

The applicability of the **PaX** fluorophores was further investigated. To show that the **PaX** fluorophores are cell-permeable, a two-step labeling strategy in U2OS cells expressing a vimentin-HaloTag construct was employed (Figure 1b–d). The bicyclononane (BCN) SPIEDAC partner was incorporated into the vimentin construct treatment with HaloTag ligand-BCN (HTL-BCN), followed by labeling with the **PaX-Tz** compounds. Once the cell permeability was confirmed, a simplified labeling strategy was devised in COS-7 cells by incorporation of the unnatural amino acid *endo*-BCN-l-lysine by genetic code expansion (GCE) into an mCerulean3-vimentin construct (Figure 1e,f). This more direct labeling strategy was able to circumvent the use of the HTL-BCN ligand as well as the HaloTag protein itself, allowing direct, minimal linkage error labeling of vimentin. Used in conjunction with MINFLUX imaging, the width of vimentin filaments was measured to be 14.4 ± 4.2 nm, in agreement with published cryo-electron microscopy (EM) data (Figure 2a–e).^8^ This demonstrates the power of MINFLUX in imaging structures with fidelity approaching that of cryo-EM. For comparison, nanobody and antibody labeling strategies were also used, giving larger values for the filament thickness (21.8 ± 5.6 and 31.4 ± 7.9 nm, respectively).

While this minimal linkage error strategy will undoubtedly be adopted by groups implementing MINFLUX or other nanometer-regime imaging techniques, it remains to be seen how well the reduction in linkage error improves the image quality when used to image biological structures whose size approaches that of the resolution limit reported for MINFLUX (∼1 nm).

This synergistic use of tetrazine labeling with caging-group-free **PaX** fluorogenic fluorophores provides insights into new strategies for reducing the linkage error and background fluorescence of fluorophores used in state-of-the-art nanoscopy techniques. Overall, the future holds great potential for the development and adoption of sophisticated fluorogenic probes, revolutionizing our understanding of the biological world at the nanoscale.
